# Genetic analysis combined with 3D‐printing assistant surgery in diagnosis and treatment for an X‐linked hypophosphatemia patient

**DOI:** 10.1002/jcla.24243

**Published:** 2022-02-02

**Authors:** Jie‐Yuan Jin, Li‐Yang Zhang, Shuai Guo, Ke Tang, Lei Zeng, Rong Xiang, Jie‐Yu Liang

**Affiliations:** ^1^ Department of Orthopaedics Xiangya Hospital of Central South University Changsha China; ^2^ School of Life Sciences Central South University Changsha China; ^3^ Hunan Key Laboratory of Animal Models for Human Diseases Central South University Changsha China; ^4^ Department of Neurosurgery Xiangya Hospital Central South University Changsha China; ^5^ Hunan Key Laboratory of Medical Genetics School of Life Sciences Central South University Changsha China

**Keywords:** FGF23, *PHEX*, three‐dimensional printing, treatment, X‐linked hypophosphatemia

## Abstract

**Background:**

Hypophosphatemia is mainly characterized by hypophosphatemia and a low level of 1alpha,25‐Dihydroxyvitamin D2 (1,25‐(OH)_2_D2) and/or 1alpha,25‐Dihydroxyvitamin D3 (1,25‐(OH)_2_D3) in the blood. Previous studies have demonstrated that variants in *PHEX* and *FGF23* are primarily responsible for this disease. Although patients with variants of these two genes share almost the same symptoms, they exhibit the different hereditary pattern, X‐link dominant and autosome dominant, respectively. Three‐dimensional (3D) printing is a method which can accurately reconstruct physical objects, and its applications in orthopedics can contribute to realizing a more accurate surgical performance and a better outcome.

**Methods:**

An X‐linked hypophosphatemia (XLH) family was recruited, with four patients across three generations. We screened candidate genes and filtered a duplication variant in *PHEX*. Variant analysis and co‐segregation confirmation were then performed. Before the operation of our patient, a digital model of our patient's leg had been rebuilt upon the CT scan data, and a polylactic acid (PLA) model had been 3D‐printed.

**Results:**

A novel duplication *PHEX* variant c.574dupG (p.A192GfsX20) was identified in a family with XLH. Its pathogenicity was confirmed by the co‐segregation assay and online bioinformatics database. The preoperative plan was made with the help of the PLA model. Then, arch osteotomy and transverse osteotomy were performed under the guidance of the previous simulation. The appearance of the surgical‐intervened leg was satisfactory.

**Conclusions:**

This study identified a novel *PHEX* variant and showed that 3D printing tech is a very promising approach for corrective osteotomies.

## 1 INTRODUCTION

Hereditary hypophosphatemia is mainly characterized by hypophosphatemia and a low level of 1alpha,25‐Dihydroxyvitamin D2 (1,25‐(OH)_2_D2) and/or 1alpha,25‐Dihydroxyvitamin D3 (1,25‐(OH)_2_D3) in the blood. This disease can be roughly divided into four main types: X‐linked dominant hypophosphatemia (XLH, OMIM 307800), autosomal dominant hypophosphatemic rickets (ADHR, OMIM 193100), autosomal recessive hypophosphatemic rickets (ARHR1, OMIM 241520; ARHR2 OMIM 613312), and X‐linked recessive hypophosphatemic rickets (OMIM 300554).^1^ The incidence rate of XLH is 1/20000 worldwide, and patients with XLH present various bone deformities, including genu varum and windswept lower limbs, which can be observed at a very young age.^2^ XLH was first discovered as a subtype of rickets refractory to vitamin D therapy, whereas vitamin D treatment is effective in most cases of rickets. Previous studies had identified the connection between XLH and phosphate‐regulating endopeptidase homolog X‐linked (PHEX).^3^ ADHR is another type of hereditary hypophosphatemia caused by mutated fibroblast growth factor 23 (FGF23).^1^ Variants of dentin matrix acidic phosphoprotein 1 (*DMP1*) and ectonucleotide pyrophosphatase/phosphodiesterase 1 (*ENPP1*) are the main causative factors of ARHRs.^4^, ^5^ Chloride channel 5 (*CLCN5*) is associated with X‐linked recessive hypophosphatemic rickets.^6^ In addition, variants of solute carrier family 34 member 1 (*SLC34A1*), family with sequence similarity 20, member C (*FAM20C*), solute carrier family 34 member 3 (*SLC34A3*), cytochrome P450 family 27 subfamily B member 1 (*CYP27B1*), and cytochrome P450 family 2 subfamily R member 1 (*CYP2R1*) were reported to trigger the hypophosphatemia phenotype.^7^, ^8^, ^9^ PHEX, a 749‐amino‐acid endopeptidase, can regulate FGF23, and FGF23 accumulation in serum decreases the reabsorption of phosphate in the renal proximal tubule, suppresses activation, and stimulates catabolism of vitamin D.^3^

Three‐dimensional (3D) printing is a method that can accurately reconstruct physical objects. It is usually applied in maxillofacial and dental surgery. However, it had been used in orthopedic surgery in recent years and achieved promising results. For example, corrective osteotomies used to be extremely complex, still, with the assistance of an accurate 3D printing model, and operations can be well performed and even virtually simulated, thus leading to a better outcome.^10^

This study reported a family with four patients with XLH across three generations. Genetic analysis was performed on the proband and her parents to clarify their hereditary etiology. A novel *PHEX* duplication variant c.574dupG (p.A192GfsX20) was identified, which was never reported. Moreover, 3D printing was employed preoperatively, helping the surgeon to visualize the anatomy in full 3D and plan the corrective osteotomy.

## 2 MATERIALS AND METHODS

### 2.1 Genetic analysis

The Review Board of Xiangya Hospital of the Central South University has approved this research. Written informed consents were collected from all family members. Genomic DNA was extracted from the peripheral blood of subjects using a DNeasy Blood & Tissue Kit (Qiagen). Primer pairs of *PHEX* (NM_000444.4) and *FGF23* (NM_020638.2) were generated, and polymerase chain reaction (PCR) was used to amplify all exons of the *PHEX* and *FGF23* (Table S1). Direct sequencing of purified PCR products was determined using the ABI 3100 Genetic Analyzer (ABI). Sequences were analyzed using DNAMAN Software (Version 8, Lynnon Biosoft) to compare our sequencing results with the reference sequence. The pathogenicity and conservation of variants were predicted by MutationTaster (http://www.mutationtaster.org/), PolyPhen‐2 (http://genetics.bwh.harvard.edu/pph2/), and SIFT (http://provean.jcvi.org/index.php). Co‐segregation was applied based on the variant identified by Sanger sequencing.

### 2.2 3D‐model reconstruction

To model the patient's left extremity, her leg was scanned using a 64‐row helical CT machine with 0.625‐mm slice thickness as previously described.^10^ The scan data were output as the Digital Imaging and Communication in the Medicine standard format. CT data were read using Boholo surgical simulator software (Boholo Medical Science), and a digital 3D model was rebuilt upon this data. The preoperative surgical plan is made, and a virtually simulated corrective osteotomy is performed on a 3D‐printed polylactic acid (PLA) model.

## 3 RESULTS

### 3.1 Case description

**FIGURE 1:**
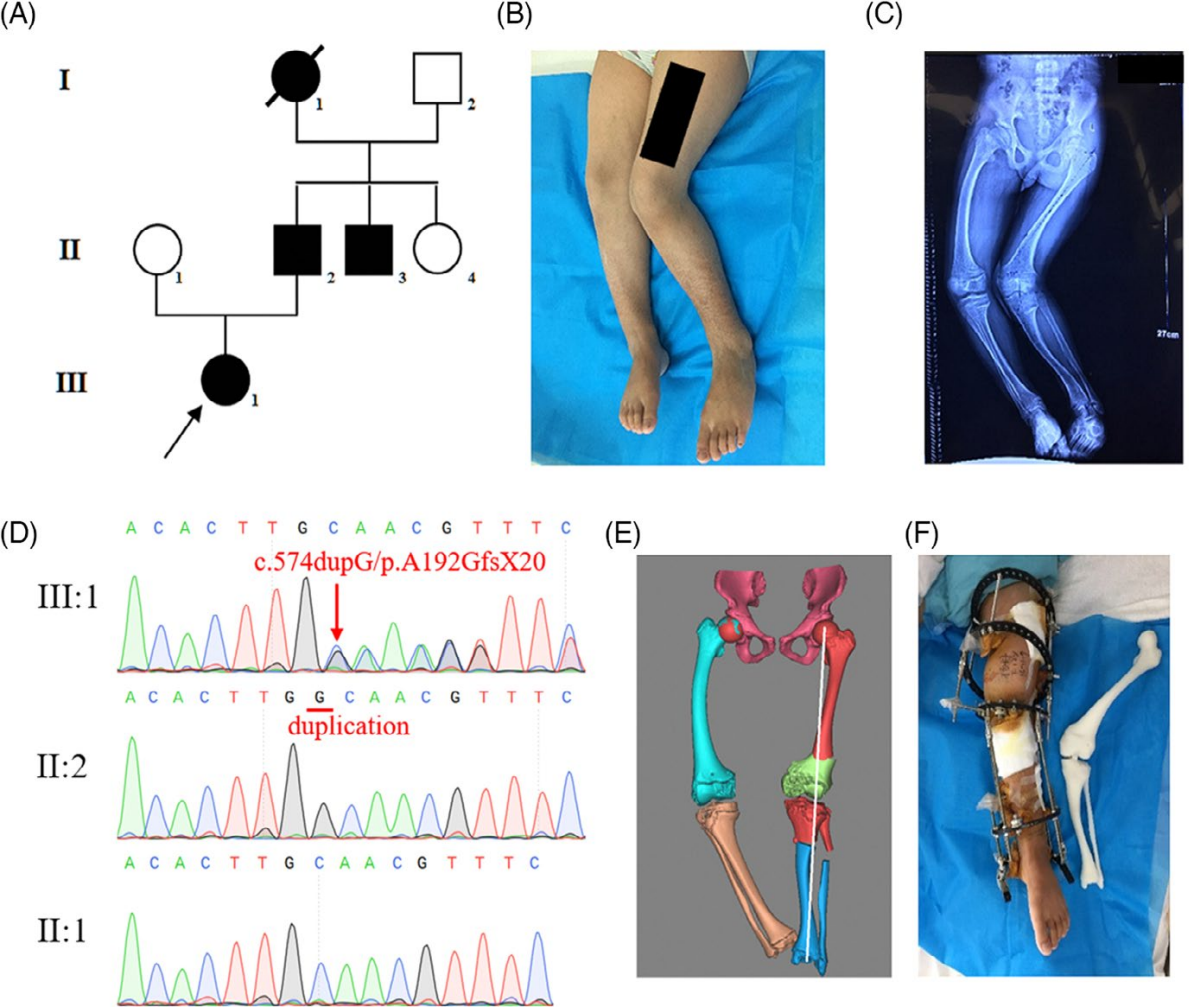
Clinical information of affected patients. (A) Pedigree of the proband's family. The proband (III‐1) and her father (II‐2) exhibited same symptoms. Squares indicate male family members; circles, female members; closed symbols, affected members; open symbols, unaffected members; arrow, proband. (B) Windswept lower limbs of the proband. (C) X‐ray image of the proband. (D) Sequencing results of the proband and her parents. The proband carried a heterozygous c.574dupG of *PHEX* exon 5, which was confirmed by reverse sequencing. The proband's father was a hemizygote of this duplication variant. The proband's mother was unaffected by this variant. (E) A digital 3D model of the bilateral lower extremities of the proband was rebuilt based on the CT scan data. The cutting angle and positions of osteotomy were verified using this virtual model. (F) A PLA model of the bone and joint was printed, and the corrective surgery was performed under the guidance of this PLA model. The appearance of the surgically intervened leg was satisfactory

A XLH family with four patients across three generations was recruited in this study (Figure 1A). The proband (III:1), a 12‐year‐old girl from the Central‐South China, was brought to our department for the orthomorphia of lower limbs. She was first diagnosed with familial hypophosphatemic vitamin D‐resistant rickets at 6 years in the local hospital, and her symptoms mainly presented gait abnormality and ever‐progressive windswept lower limb deformities (Figure 1B,C). The proband also showed a mildly elevated serum parathyroid hormone level. Her serum 25‐hydroxy vitamin D concentration was less than normal (7.48 ng/mL), along with normal serum calcium and phosphate levels (Table 1). Moreover, her father (II:2) had rickets with genu varum. Two other family members had rickets (I:1 and II:3), but their blood samples were unavailable.

**TABLE 1:** Lab test results of the proband

Test name	Results	Reference range
PTH	93.6 pg/mL	15–65 pg/mL
25‐OHVD	7.48 ng/mL	>20 ng/mL
hCT	<0.50 pg/mL	0–9.82 pg/mL
Potassium	4.15 mmol/L	3.5–5.3 mmol/L
Sodium	138.7 mmol/L	137–147 mmol/L
Chloride	103 mmol/L	99–110 mmol/L
CO_2_CP	22.8 mmol/L	19–33 mmol/L
AG	12.9 mmol/L	8–16 mmol/L
Calcium	2.34 mmol/L	2–2.6 mmol/L
Serum phosphorus	0.97 mmol/L	0.86–1.78 mmol/L
Magnesium	0.84 mmol/L	0.66–1.07 mmol/L

Abbreviations: 25‐OHVD, 1, 25‐(OH)_2_ Vd3, cholecalciferol; AG, anion gap; CO_2_CP, carbon dioxide combining power; hCT, human calcitonin; PTH, Parathyroid hormone.

### 3.2 Variant analysis

The known causative genes *PHEX* and *FGF23* were examined in all available family members to elucidate the genetic mechanism of their XLH. After Sanger sequencing, a novel duplication variant of *PHEX* c.574dupG (p.A192GfsX20) was detected in the proband and her father (Figure 1D). Her mother did not harbor this variant. This newly identified variant was neither found in other unaffected family members, nor reported in GnomAD (http://gnomad.broadinstitule.org). Its pathogenicity was confirmed by the co‐segregation assay and online bioinformatics database. Adherence to the standards and guidelines of the American College of Medical Genetics and Genomics (ACMG), we classified the *GATA5* variant as “Pathogenic” (Table 2).^11^ Thus, it was concluded that this variant could be the genetic etiology of XLH in this family.

**TABLE 2:** *PHEX* variant identified in this study and its pathogenicity prediction and classification

Gene	Variant	MutationTaster	PolyPhen‐2	SIFT	GnomAD	CMDB	OMIM clinical phenotype	American College of Medical Genetics classification
*PHEX*	NM_000444.4: c.574dupG (p.A192GfsX20)	D	‐	‐	‐	‐	XLD, Hypophosphatemic rickets, X‐linked dominant	Pathogenic (PVS1, PM2, PP1, PP3)

Abbreviations: D, disease‐causing; XLD, X‐linked autosomal dominant.

### 3.3 Surgical reconstruction

The preoperative examination of the proband showed normal myodynamia and muscular tension. As traction therapy might lead to stiffness of the knee, corrective surgery was performed on the patient. The 3D printing technique was used to rebuild her deformed left femur and tibiofibular structures *ex vivo* as previously described.^10^ A PLA model was 3D‐printed to verify cutting angle and positions of the corrective osteotomy (Figure 1E). Then, the arch osteotomy of the left femur bone and transverse osteotomy of the left upper tibiofibular were performed under the guidance of the previous simulation. After the operation, the patient's 35‐degree left genu varum was repaired, and her left leg was then fixed using an Ilizarov external fixator (Figure 1F). The appearance of the surgically intervened leg was satisfactory. The long‐term effect needed further follow‐up observation.

## 4 DISCUSSION

In this study, a novel duplication *PHEX* variant c.574dupG (p.A192GfsX20) was identified in a XLH family with four patients across three generations. According to the standards and guidelines of ACMG, we classified the duplication variant in *PHEX* as “Pathogenic”: (1) Loss‐of‐function variants in PHEX had been confirmed to trigger XLH, and the present variant was a duplication variant, which can lead to a truncated PHEX protein (PVS1). (2) The variant was not in controls (data from GnomAD and CMDB database, an authoritative database concentrating on the variant frequency in the Chinese population; PM2). (3) Bioinformatics software (MutationTaster, PolyPhen‐2, and SIFT) predicted the variant to be disease‐causing (PP3).^11^ Thus, we reasoned that the *PHEX* variant was associated with XLH in this family.

*PHEX* encodes a 749‐amino‐acid endopeptidase that is vital in the reabsorption of phosphate in the proximal renal tubule and in vitamin D production.^3^ Variants in *PHEX* can disrupt its binding to FGF23 and then lead to FGF23 accumulation. FGF23 has been demonstrated to be able to inhibit the phosphate intake of renal epithelial cells lining the proximal tubule.^3^ PHEX includes three functional domains: a short intracellular domain, a single transmembrane domain, and a large extracellular domain.^12^ This novel duplication variant was located at the beginning of the extracellular domain. The bioinformatics analysis predicted this site to be highly evolutionarily conserved (Figure S1). Moreover, this frameshift variant of *PHEX* would cause a premature stop codon, which not only results in a truncated protein, but also, according to nonsense‐mediated mRNA decay theory, leads to the decreased levels of *PHEX* mRNA in affected patients.^13^ Other small insertions, such as c.466_467insAC (p.L156HfsX66) and c.682dupT (p.S228FfsX10), are located upstream or downstream from this site had been reported to be pathogenic.^14^ The symptoms of the proband were consistent with previous studies in terms of PHEX loss.^15^ Thus, it was reasoned that this duplication variant was the main underlying pathogenic factor in the present case. Patients with XLH can be misdiagnosed as other types of rickets with only biochemical tests because the rickets symptoms are not specific.^16^ Moreover, XLH is more refractory than the standard nutritional rickets treatment. The misdiagnosed patients would develop bone deformities that have to be surgically corrected due to delayed treatment. The genetic analysis in *PHEX* and *FGF23* contributes to accurately diagnosing the patients with hereditary hypophosphatemia. Severity of the disease is similar in males and females, although XLH is an X‐linked disease, in which a gene dosage effect would lead to milder symptoms in female cases, in theory. In the present study, the phenotype of lower limbs in the proband was not milder than her father.^17^

Patients with XLH can be observed with knee deformities. However, whether the untreated malformations lead to osteoarthritis and joint pain is still unclear. In the present study, the left lower extremity was repaired first because it presented more severe deformities. Orthopedic surgery will be performed on the right leg later. Further, the 3D printing technique was used to rebuild the deformed left femur and tibiofibular structures *ex vivo* before performing an operation. The application of this technique in orthopedics and plastic surgery was started recently because it could construct *ex vivo* models to aid preoperative evaluation and effectively promote operational accuracy.^10^ Moreover, during the operation, the Ilizarov external fixator was used rather than internal fixation because the operation site was quite close to epiphysis and hence internal fixation might interfere with epiphysis, disrupting bone development. Transverse osteotomy and slow re‐correction were employed to avoid iatrogenic common peroneal nerve injury, assisting in the correction of remaining malformations.

Except orthomorphia, we recommended the combined treatment of phosphates (5 times/day with ingestion of 30 mL every time) and Rocaltrol (0.5 μg/day) for patients in the family.^18^ Unfortunately, the proband and her father were not provided the timely medical intervention, which resulted in severe skeletal deformities. It is challenging to confirm hypophosphatemia in infants and easily misdiagnosed as hypovitaminosis.^19^ Genetic screening (especially *PHEX* and *FGF23* variant screening) contributes to clinical diagnosis of hypophosphatemia.

This study reported a novel variant of *PHEX* in a family of patients with XLH, which enriched the database of *PHEX* variants in the Chinese population. The sequencing of XLH‐related gene in patients might benefit future investigation into the genesis of XLH and functions of protein PHEX and FGF23. The 3D printing technique promoted the operation accuracy.

## CONFLICT OF INTEREST

The author(s) declared no potential conflicts of interest with respect to the research, authorship, and/or publication of this article.

## AUTHOR CONTRIBUTIONS

Jie‐Yuan Jin and Li‐Yang Zhang contributed to conception and design and carried out analysis and interpretation of data; Shuai Guo and Lei Zeng performed acquisition, analysis, and interpretation of data; Ke Tang carried out analysis and interpretation of data; Jie‐Yu Liang contributed to conception and design and wrote the original draft; Rong Xiang revised the draft and finally approved the version.

## Supporting information

Figure S1

Table S1

## Data Availability

The datasets used and/or analyzed during the current study are available from the corresponding author upon reasonable request.
