# Supplementary material for: Genetic analysis combined with 3D‐printing assistant surgery in diagnosis and treatment for an X‐linked hypophosphatemia patient
Source: J Clin Lab Anal. 2022 Feb 2;36(3):e24243. doi: 10.1002/jcla.24243 (PMC8906030; doi:10.1002/jcla.24243)
Supplement: Supplementary file 1 — Figure S1 [file JCLA-36-e24243-s001.zip › jcla24243-sup-0001-FigureS1.docx]

**Figure legends**

**Figure S1.** These amino acid sites were highly evolutionarily conserved cross-species.
