# Supplementary material for: Genetic analysis combined with 3D‐printing assistant surgery in diagnosis and treatment for an X‐linked hypophosphatemia patient
Source: J Clin Lab Anal. 2022 Feb 2;36(3):e24243. doi: 10.1002/jcla.24243 (PMC8906030; doi:10.1002/jcla.24243)
Supplement: Supplementary file 2 — Table S1 [file JCLA-36-e24243-s002.docx]

| **Table S1. PCR Primers for Directed Sequencing Analysis of *PHEX* and *FGF-23*** | | | |
| --- | --- | --- | --- |
| **Gene** | **Exon** | **Forward Primer (5' to 3')** | **Reverse Primer (5' to 3')** |
| PHEX | 1 | GCTCTTGAGACCAGCCACCA | GCAGGCAAACAGCCCTATACC |
| PHEX | 2 | CTTGCGTATGTTTCCGAGGG | ACCTGTCTTCTCTTCCACTTCCC |
| PHEX | 3 | AGGCTTGGAAACTGGTTGA | GTCATGCTTCAAATCCCAAA |
| PHEX | 4 | CTGGAGGTTGGAATTGTGAT | TTCTCCACCAAGCCAGTAA |
| PHEX | 5 | TGATTATGCTCATCCCACCC | AAGCCGGGAGAAGGGAATA |
| PHEX | 6 | CTGGGATGCAGACGATTTCA | TAGCCACACTGCTCATTTCC |
| PHEX | 7 | TATGGGTGCCTGGTATTGC | CAGGGAGGTGGAAGGAATG |
| PHEX | 8 | GGACCACACCAAAGCCTTGA | GCATCCCAATACACAGACAAGGG |
| PHEX | 9 | CAGAGGTAACAGTGAGCCAAGA | AAGGATGTGAGAAGGGAAGC |
| PHEX | 10 | GTCCCTCGATGGAGCTTTG | CAATAGAGCTTGGGCTACAA |
| PHEX | 11 | ATGGGTTAGGGTGTGCAGTGTT | CACCTGGAAGGCTGACATTAGC |
| PHEX | 12 | TGGGCCTGGTTAGTTATCTTT | AGGAAAGGCCGAATTACAAG |
| PHEX | 13 | GAAGGGCGCATTTCTACAT | TACGCATCGTTTCTGACACTT |
| PHEX | 14 | AGTTGCTCCTTCCTATGCTG | GACTCCGCTTCTCACCAAT |
| PHEX | 15 | CCCCTCATGTCCAACATCCC | TTCCTTCACCAGCATACCCTG |
| PHEX | 16 | CAGGAGGAGTGCCTTTCAGATG | ACCCTGGTAACAAGGATCAGAAAAC |
| PHEX | 17 | GCAGTTTATCTTGGCTTTCC | AAGCCATCACAGCAAGACACG |
| PHEX | 18 | GTGAGGGAAAGGAAAGATGA | TGAGGCATAGCAAGGATTTA |
| PHEX | 19 | ATTACATTCCAGAGCACCTT | CATGGCTATGGTATGAATTGAGG |
| PHEX | 20 | GCAAAGAGAAAAACCCACCG | GATCAAGGGAGCAAACTCAA |
| PHEX | 21 | ATACACTGGTCGATTCCTGG | TCACTGGCACTTGATGTAGC |
| PHEX | 22 | TTTTGGGCTTTAGTTGTCTCC | GCATTCCACAGAGCAGCAA |
| FGF-23 | 1 | GGATGTGGACAGTGGAGTTT | GAAAGCTAGGAGGGTTGGATTAG |
| FGF-23 | 2 | GCAAGAGAGGATGTCAGAAGAA | CAATGGGCAGTGCAGACTA |
| FGF-23 | 3 | GTCTCTGAAAGGGCGAACTAT | TGCTGAGGGATGGGTTAAAG |
